# A prospective cohort study of disability pension due to mental diagnoses: the importance of health factors and behaviors

**DOI:** 10.1186/1471-2458-13-621

**Published:** 2013-07-02

**Authors:** Åsa Samuelsson, Annina Ropponen, Kristina Alexanderson, Pia Svedberg

**Affiliations:** 1 Division of Insurance Medicine, Department of Clinical Neuroscience, Karolinska Institutet, Stockholm SE-171 77, Sweden; 2 School of Medicine, University of Eastern Finland, Kuopio, Finland; 3 Finnish Institute of Occupational Health, Helsinki, Finland

**Keywords:** Sick leave, Disability pension, Mental disorders, Self-rated health, Life-style, Twins

## Abstract

**Background:**

Previous studies have found associations between various health factors and behaviors and mental disorders. However, knowledge of such associations with disability pension (DP) due to mental diagnoses is scarce. Moreover, the influence of familial factors (genetics and family background) on the associations are mainly unknown. The aim of the study was to investigate associations between health factors and behaviors and future DP due to mental diagnoses in a twin cohort, accounting for familial confounding.

**Methods:**

A prospective cohort study of Swedish twins (N=28 613), including survey data and national register data on DP and other background factors was conducted. Cox proportional hazards regression models were used to calculate hazard ratios (HR) with 95% confidence intervals (CI) for the whole twin cohort, and for discordant twin pairs.

**Results:**

During follow-up 1998–2008 (median 10 years), 2.2% of the cohort was granted a DP with a mental diagnosis. In the fully adjusted analyses of the whole cohort, the associations of poor or moderate self-rated health (SRH), under- or overweight, former or current tobacco use, or being an abstainer from alcohol were significantly associated with risk of DP due to mental diagnoses. Analyses of discordant twin pairs confirmed all these associations, except for current tobacco use, being independent from familial confounding. Exclusion of individuals with current or previous depression or anxiety at baseline did not influence the associations found.

**Conclusions:**

Poor or moderate SRH, under- or overweight, former tobacco use or being an abstainer from alcohol seem to be strong direct predictors of DP due to mental diagnoses, independently of several confounders of this study, including familial factors.

## Background

Mental disorders, alongside musculoskeletal disorders, are today the major disability pension (DP) diagnostic groups in OECD countries [1]. Even though the decision to grant a DP due to a mental diagnosis is based on a medically confirmed mental disorder, a DP is a multi-factorial phenomenon, being influenced by several factors, including subjective health and health behaviors (weight, physical activity, tobacco use, and alcohol consumption) [2,3]. Although the associations of self-rated health (SRH) [4,5] and health behaviors [6-12] with the risk of mental disorders are well established, there is little knowledge of the associations of SRH and health behaviors with the direct risk of being granted a DP due to a mental diagnosis [13,14]. More knowledge of influential health-related factors to the risk of DP due to mental diagnoses, might provide a basis for identifying risk groups and implement preventive measures [2,14].

Studies of DP in general, and of DP due to mental diagnoses, have shown that poor/moderate SRH, in comparison with excellent/good SRH, is associated with a higher risk of DP, even after accounting for various confounders [15-22]. However, since some of these studies have only included men [15-17], and/or used rather selective samples, such as certain occupational groups [15-18], there is a need for large population-based investigations, including both sexes, to further examine the associations found.

Studies of the association between leisure-time physical activity and DP in general have found a protective effect of high physical activity on DP [19,21-27]. Also, a study based on Swedish twins has found that being consistently sedentary or decreasing physical activity between two time points is a risk factor for DP in general [28]. For DP due to mental diagnoses, we are only aware of one study investigating the association of leisure-time physical activity with risk of such DP [27], with similar findings as for DP in general.

Underweight or overweight/obesity has been shown to be associated with increased risk of DP in general, in comparison with normal weight [28-31]. Similar findings for DP due to mental diagnoses have been found for men [32-34]. However, in a recent study, based on both male and female Finnish municipality workers [31], no significant associations between BMI and DP due to mental diagnoses could be established, after accounting for various confounders. However, in that study they used other categories for under- and normal weight and obesity than recommended by the World Health Organization (WHO) [35].

Studies of smoking in relation to DP in general have shown that former or current regular smoking [19,21-23,25,26,36-38] and the steady use of tobacco products [28] are associated with risk of DP. However, only one of these studies, which investigated male construction workers, specifically studied DP due to mental diagnoses [36].

For alcohol consumption and the risk of DP in general or DP due to mental diagnoses a U-shaped relationship has been suggested; that is, both being an abstainer or infrequent consumer and being a heavy or frequent consumer seem to be associated with higher risk of DP [22,23,25,39-45].

SRH [46,47], leisure-time physical activity [48,49]; weight, as measured with the body mass index (BMI) [50,51]; tobacco use (smoking and use of moist snuff) [52]; and alcohol consumption [53] have all been found to be moderately heritable traits, as too has DP due to mental diagnoses [54,55]. This shows the importance of accounting for potential familial confounding (genetics and family background, e.g. social background) when studying the associations between SRH, health behaviors and DP due to mental diagnoses. Previous studies, based on unrelated individuals, have not been able to account for familial confounding in their associations. However, by studying twins, who to certain extent share their genetics (100% for monozygotic (MZ) and ≈50% for dizygotic (DZ) ) and all shared environmental factors (100% for both MZ and DZ), e.g. intrauterine and rearing, this is possible [56]. There are some studies of Nordic twin cohorts which have investigated the direct associations of health behaviors with DP [28,57-59]. However, none of these focused specifically on DP due to mental diagnoses.

The aims of this prospective cohort study were to investigate the associations of health factors and behaviors with DP due to mental diagnoses among women and men in a Swedish twin cohort, and to establish whether familial factors influenced the associations found. The hypotheses were that poor or moderate SRH, either underweight or overweight/obesity, low leisure-time physical activity, current or former tobacco use, and either being an abstainer from alcohol/infrequent consumer or high/frequent consumer of alcohol are independent predictors of DP due to mental diagnoses.

## Methods

A prospective twin cohort study was performed, using data from the Swedish Twin Study of Disability Pension and Sickness Absence (STODS) [60].

### Study population and data

STODS includes all twins born 1925–1958 in Sweden (29 799 complete twin pairs) and identified in the Swedish Twin Registry (STR) [61]. The STR provided data on date of birth, sex, pair identification, and zygosity, and self-reported survey data were available for SRH, health behaviors, diseases, major depression (MD), and generalized anxiety disorder (GAD). The latter information was collected through telephone interviews in the ‘Screening Across the Lifespan Twin Study (SALT)’, carried out between January 1998 and March 2003 [61].

To include information on DP, censoring, and background factors, data from national registries were linked to all the twins, using the unique personal identification number assigned to all people living in Sweden. Information on DP (date and diagnosis) was obtained from Sweden’s National Social Insurance Agency. DP due to mental diagnoses were coded according to the International Classification of Diseases (ICD), F-chapter (F00-F99) [62]. Information on educational level, marital status, old-age pension, and emigration was obtained from Statistics Sweden, and dates of death were obtained from the Cause of Death Registry, maintained by the National Board of Health and Welfare.

To be eligible for this study, the twins needed to have participated in the SALT study (n=39 261; 66%). Other inclusion criteria were that the individuals, at the time of the SALT study, were living in Sweden, below the age of 65, and neither on DP nor old-age pension. The final sample consisted of 28 613 individuals (52% women), of which there were 10 531 complete twin pairs (2811 monozygotic (MZ), 3783 dizygotic (DZ), 3835 opposite-sexed DZ, 102 unknown zygosity) and 7551 single individuals without information on the co-twin due to non-participation in the SALT-interview, death, emigration, DP, or early old age pension. The twins were between 41 and 64 years of age (mean 52.9, SD 5.6) at inclusion. During follow-up, information was obtained about the individuals on person time in days until death, emigration, reaching 65 years of age, or the granting of DP or old-age pension (whichever came first) up to the end of follow-up (31^st^ of December 2008).

### Social insurance

All citizens in Sweden between the ages of 16 to 64 are covered by the national public social insurance scheme managed by the National Social Insurance Agency. There are two prerequisites for having the right to be granted DP; the individual has to have a medically confirmed disease or injury, and that disorder must permanently restrict his or her working work capacity, basically in all type of jobs. Based on a thorough assessment of level of work incapacity, the Social Insurance Agency can grant DP. DP covers about 65% of lost income [63]. If the individual has not had income before, a basic sum is granted. For this study, the outcome was defined as being granted a DP due to mental diagnoses during follow-up 1998–2008.

### Health factors and behaviors

*SRH* was assessed using the following question:“*How would you rate your general health status*?”, with five response options: ‘*excellent*’, ‘*good*’, ‘*moderate*’, ‘*fairly poor*’, and ‘*poor*’. For this study, the two upper and the two lower groups were combined, and the excellent/good group was used as the reference category [64]. SRH has proven to be a reliable measure of future morbidity and mortality [4].

*Leisure*-*time physical activity* was assessed using the question: “*If you consider the physical activity you take during your leisure time*, *which of these seven options fit you the best if you look at the year as a whole*?”. The response options ranged from '*very much*' to '*almost never*'. For this study, the separate options were grouped into three categories, namely: high ('*very much*’/'*a lot*'), moderate ('*quite a lot*', '*not much*', '*very little*'), and low ('*hardly ever*'/'*almost never*'). The moderate group was used as the reference category [49]. *BMI*, which has been shown to be a reliable measure of underweight/overweight, was calculated by dividing weight in kilograms by height in meters squared [65,66]. As an exposure variable, BMI was divided into four groups according to WHO criteria’s [35]: underweight (<18.5), normal weight (18.50-24.99), overweight (25.00-30.00), and obesity (>30.00). As a covariate, BMI was used as a continuous variable. *Tobacco use*, including the use of both cigarettes and moist snuff, was measured in four categories: ‘*never tried*’ (never tried, including only tried), ‘*current user*’ (occasional or regular), and ‘*former user*’. Never tried was used as the reference category [28]. *Alcohol consumption* was measured in two ways. Those who either responded yes to '*ever been drinking alcohol*' or no to '*drinking alcohol the last two months*' were asked probing questions about the total alcohol (beer, wine, or liquor) they consumed on a typical occasion (infrequent consumption). Those who, on the other hand, responded yes to '*drinking alcohol the last two months*' were asked probing questions about the total alcohol (beer, wine, or liquor) they consumed on weekdays (Monday to Thursday) and at weekends (Friday to Sunday) (frequent consumption). Beer was measured in glasses/bottles (ranging from one glass to 6 bottles or more), wine in glasses/bottles (ranging from one glass to one bottle or more), and liquor in centiliters (ranging from 4 to 37 centiliters or more). The different units were then converted into grams, using the Alcohol Use Disorders Identification (AUDIT) guidelines [67], as adapted for use in Sweden. According to AUDIT, a standard drink, e.g. a glass of wine, contains 12 grams of pure alcohol [67,68]. For the group of infrequent consumers, the cut-off point for heavy consumption was set at 48 grams/occasion for men and 36 grams/occasion for women; for light consumption at 12 grams of alcohol/occasion for both men and women, and for moderate consumption, at 13 to 48 grams/occasion for men and 13 to 36 grams/occasion for women, according to current Swedish recommendations [68]. For the group of frequent consumers, the cut-off point for heavy consumption was set at 168 grams/week for men and 108 grams/week for women; light consumption was defined as 12 to 84 grams alcohol/week for both men and women, and moderate consumption as 85 to 168 grams/week for men and 85 to 108 grams/week for women [68]. Finally, those who reported no consumption of alcohol were considered as abstainers. Light frequent alcohol consumption was used as the reference group [41].

### Background factors

Male *sex* was used as the reference group. *Age* at interview was measured as a continuous (birth year) variable. *Education* was measured as a continuous variable (years of study). *Marital status* was dichotomized into married (reference group) and unmarried (non-married, divorced, widow/widower). Both education and marital status were measured in the year 2000. *Severity of diseases* was measured by applying the rating schema devised by Gold and colleagues [69], slightly revised by Svedberg et al. [64] (Table 1), to the data on different diseases. Accordingly, diseases were rated as ‘*very life threatening*’, ‘*somewhat life threatening*’, or ‘*not at all life threatening*’. If an individual had several diseases with varying degrees of severity, the most severe disease was used. ‘No disease reported’ was used as the reference group. Current or previous history of *MD* was measured using WHO’s Composite International Diagnostic Interview procedure [70], which involves conducting a short diagnostic interview to measure mental disorders according to Diagnostic Statistical Manual of Mental Disorders, version IV, (DSM-IV) [71] and ICD, version 10 [62]. People were considered as having a MD if they screened positive on the initial question “*In your lifetime*, *have you ever had two weeks or more when you nearly every day felt sad*, *blue*, *depressed*?”, and on at least four out of eight additional symptoms: ‘*lost interest* (*in general and*/*or in hobbies*)’, ‘*changed weight* (*gained or lost*)’, ‘*trouble falling asleep*’, ‘*feeling tired*, *trouble concentrating*’, and/or ‘*thoughts about death*’. Current or previous history of *GAD* was measured according to DSM-IV [71]. People were considered as having a GAD if they screened positive on the initial question "*Have you had an episode lasting at least a month in which you felt worried and anxious most of the time*?", and on least one out of five additional symptoms: ‘*feeling tense*’, ‘*irritable*’, *tired*, ‘*trouble sleeping*’, and the combination of ‘*being restless*’ and ‘*on the edge*’.

**Table 1:** **Medical diseases categorized according to being considered life-threatening or not**^**1**^

**Very life-threatening**	**Somewhat life-threatening**	**Not at all life-threatening**
Angina pectoris	Allergies and Asthma	Back disorders
Cancer	Cardiac arrhythmia	Cataract
Cardiac insufficiency	Cardiac murmur	Dizziness
Heart attack	Chronic bronchitis	Eczema
Multiple sclerosis	Diabetes	Gall disorders
Stroke	Dyslipedimia	Glaucoma
Thrombosis in leg	Emphysema	Gout
	Epilepsy	Knee and hip disorders
	Goiter disorders	Migraine
	Hay fever	Neck and shoulder disorders
	High blood pressure	Other eye disorders
	Kidney disease	Psoriasis
	Liver disease	Sciatica
	Mental complaints	Scoliosis
	Osteoporosis	Stomach and intestinal disorders
	Parkinson's disease	Urinary tract disorders
	Physical disability	
	Polio	
	Prostate complaints	
	Rheumatoid arthritis	
	SLE (Lupus)	
	Tuberculosis	
	Vascular spasms in legs	

^1^Based on the categorizations made by Gold et al. [69], modified by Svedberg et al. [64].

### Statistical analyses

First, descriptive statistics (percentages, means, medians and correlations) were calculated. The correlation coefficients between the health factors and behaviors were all below 0.30 (range 0.05-0.30), so all the factors were considered to be acceptable included in the models. Then, Cox proportional hazard regression models were applied to the whole cohort, and hazard ratios (HR) with 95% confidence intervals (CI) were computed. The underlying time scale was time since interview in the study, and the time units were days. All the self-reported variables had internal missing (1-6%) due to non-participation in a single item or a non-response to a question as a whole (don’t know or don’t want to answer). In order to include the maximum number of individuals in the statistical analyses, the missing information for each variable was coded in a separate category. Hence, the missing category contributed to variation in the analyses, but was not interpreted in the results. However, for the continuous covariates, years of education and BMI, missing observations (n=95 and 431, respectively) were not included in the analyses. The proportional hazards assumption was tested by graphically plotting all the categorical variables; all the curves were found to be parallel. In order to investigate potential sex differences, interaction terms between sex and the studied exposures were added in the models and evaluated with likelihood ratio test. However none of these tests turned out to be significant why we decided to combine men and women in the further analyses, although adjusting for sex. Hence, the first (base) model was adjusted for age and sex, the second model additionally for education, marital status, and severity of diseases, and the third model additionally for health behaviors, including leisure-time physical activity, BMI, tobacco, and alcohol, depending on the factor under study. Finally, SRH was added to complete the full model. All analyses were corrected for within-pair dependency, by clustering on pair identification, to avoid underestimation of the variance. In an attempt to account for the potential confounding effects of current or previous history of MD and/or GAD at baseline, we performed additional analyses from which these individuals were excluded (n=4615) (data not shown). The estimates were in the same direction and of the same magnitude as those for the whole cohort.

Second, conditional Cox proportional hazard analyses were applied to complete MZ and same-sexed DZ twin pairs who were discordant with regard to DP. By stratifying the analyses by pair identification, the follow-up time to DP of a twin was analyzed in relation to the follow-up time of the co-twin. The purpose of this discordant twin pair analysis was to control for age, sex, genetics (100% for MZ and≈50% for DZ), and shared environmental factors, such as intrauterine and rearing environment (100% for both MZ and DZ) [56]. First, the twin pairs were analyzed together to study the influence of the combined effect of genetics (50-100%) and shared environment (100%) for the associations. If an association between an exposure and DP in the total cohort attenuates in the analyses of discordant MZ and DZ twin pairs, familial factors may confound that association. However, if the association remains, other factors not shared by the twins, e.g. adulthood choices may explain the association. Second, the analyses were stratified by zygosity group in order to study the separate effects of genetics and shared environment for the associations. If an association attenuates among both MZ and DZ discordant twin pairs this would indicate presence of familial confounding, mainly from shared environment. If, on the other hand, the association remains, although somewhat lowered, among discordant DZ twins, but attenuates among discordant MZ twins this might indicate the presence of familial confounding, mainly from genetic factors [56]. In total, 229 twin pairs were discordant for DP due to mental diagnoses, of which 95 were MZ and 134 DZ twin pairs. All statistical analyses were carried out using STATA version 11 [72]. The study was approved by the Regional Ethical Review Board in Stockholm (2007/524-31).

## Results

In total, the twin individuals contributed 246 101 person-years at risk of DP during follow up 1998–2008. The median follow-up time was 10 years (inter quartile range 8–10 years), and 3073 individuals (10.7%) were granted a DP during follow-up. Among all DPs, 20.2% (n=621) were due to mental diagnoses. Depression (F30-F39) and anxiety (F40-F48) diagnoses constituted the largest sub-groups of the mental diagnoses, 48% and 37%, respectively. Among those granted DP due to mental diagnoses, 72% were women. Mean age at the time of being granted DP due to a mental diagnosis was 53 years (SD 4.6) (Table 2).

**Table 2:** Frequencies (percentages) for background factors and exposures in relation to different groups during follow-up

**Background factors**	**Total cohort n=28 613 (100%)**	**No disability pension n=25 540 (89%)**	**Disability pension -other diagnoses n=2452 (9%)**	**Disability pension - mental diagnoses n=621 (2%)**
**Demographics**				
**Sex**				
Men	13 767 (48)	12 630 (49)	960 (39)	177 (28)
Women	14 846 (52)	12 910 (51)	1492 (61)	444 (72)
**Zygosity**				
MZ	7084 (25)	6340 (25)	594 (24)	150 (24)
DZ same-sexed	10 431 (36)	9338 (37)	882 (36)	211 (34)
Unknown	480 (2)	421 (1)	52 (2)	7 (1)
DZ opposite-sexed	10 618 (37)	9441 (37)	924 (38)	253 (41)
**Age** (Mean; SD) range 41–64 years	52.9 (5.6)	52.7 (5.7)	54.3 (4.5)	53.0 (4.6)
**Marital status**^**1**^				
Married	18 376 (64)	16 514 (65)	1542 (63)	320 (52)
Unmarried	10 181 (36)	8972 (35)	908 (37)	301 (48)
Missing	56 (0)	54 (0)	2 (0)	0 (0)
**Education** (Mean: SD) range 8–21 years^1^	11.4 (2.6)	11.5 (2.6)	10.6 (2.2)	12.0 (2.6)
**Health factors**				
**Severity of diseases**				
No diseases reported	4739 (17)	4529 (18)	143 (6)	67 (11)
Diseases reported not at all life-threatening	7377 (26)	6825 (27)	458 (19)	94 (15)
Diseases reported somewhat life-threatening	12 664 (44)	11 049 (43)	1268 (52)	347 (56)
Diseases reported very life-threatening	3726 (13)	3038 (12)	575 (24)	113 (18)
Missing	107 (0)	99 (0)	8 (0)	0 (0)
**Major Depression**				
Yes	4205 (15)	3494 (14)	470 (19)	241 (39)
No	23 951 (84)	21 640 (85)	1947 (80)	364 (59)
Missing	457 (1)	406 (1)	35 (1)	16 (2)
**Generalized Anxiety Disorder**				
Yes	2017 (7)	1611 (6)	237 (10)	169 (27)
No	25 490 (89)	23 009 (90)	2082 (85)	399 (64)
Missing	1106 (4)	920 (4)	133 (5)	53 (9)
**Self-rated health**				
Good	21 922 (77)	20 496 (80)	1117 (46)	309 (50)
Moderate	4632 (16)	3737 (15)	734 (30)	161 (26)
Poor	1927 (7)	1189 (5)	589 (24)	149 (24)
Missing	132 (0)	118 (0)	12 (0)	2 (0)
**Health behaviors**				
**Leisure-time physical activity**				
High	2913 (10)	2640 (10)	212 (9)	61 (10)
Moderate	22 429 (78)	20 093 (79)	1866 (76)	470 (76)
Low	3009 (11)	2576 (10)	348 (14)	85 (14)
Missing	262 (1)	231 (1)	26 (1)	5 (0)
**Body mass index**				
Underweight (<18.5)	307 (1)	260 (1)	30 (1)	17 (2)
Normal weight (18.5-25)	15 371 (54)	13 902 (54)	1153 (47)	316 (51)
Overweight (25–30)	10 327 (36)	9137 (36)	967 (39)	223 (36)
Obesity (>30)	2177 (8)	1859 (7)	264 (11)	54 (9)
Missing	431 (1)	382 (2)	38 (2)	11 (2)
**Tobacco use**				
Never tried	9557 (33)	8717 (34)	664 (27)	176 (28)
Former	9995 (35)	8940 (35)	868 (35)	187 (30)
Current occasional	960 (3)	863 (3)	70 (3)	27 (4)
Current regular	6457 (23)	5524 (22)	737 (30)	196 (32)
Missing	1644 (6)	1496 (6)	113 (5)	35 (6)
**Alcohol consumption**^**2**^				
Abstainer	2588 (9)	2246 (9)	246 (10)	96 (15)
Light frequent	14 150 (49)	12 841 (50)	1041 (42)	268 (43)
Moderate frequent	2886 (10)	2655 (11)	184 (8)	47 (8)
Heavy frequent	1126 (4)	1001 (4)	103 (4)	22 (4)
Light infrequent	828 (3)	696 (3)	110 (4)	22 (4)
Moderate infrequent	3102 (11)	2665 (10)	360 (15)	77 (12)
Heavy infrequent	2783 (10)	2403 (9)	311 (13)	69 (11)
Missing	1150 (4)	1033 (4)	97 (4)	20 (3)

^1^In 2000; information on education missing for 95 individuals.

^2^Different cut-off points for men and women.

The following factors were associated with increased risk of being granted DP due to a mental diagnosis in the base model adjusted for age and sex (Table 3): moderate (HR 2.52; 95% CI 2.08-3.07) or poor (6.61; 5.39-8.11) SRH; low physical activity at leisure time (1.48; 1.18-1.87); underweight (2.45; 1.50-3.98), overweight (1.25; 1.05-1.50) or obesity (1.38; 1.03-1.84); former (1.59; 1.06-2.39) or current (1.73; 1.41-2.12) regular use of tobacco; and abstention from alcohol (2.40; 1.90-3.03). Accounting for background factors (education, marital status, and severity of diseases, in model 2), and other health behaviors (physical activity, BMI, tobacco, and/or alcohol, in model 3) had only a minor influence on the associations of SRH, leisure-time physical activity, BMI, tobacco use and alcohol consumption with DP. However, further accounting for SRH (in the full models) attenuated the associations of low physical activity (1.06; 0.84-1.36) and obesity (1.01; 0.75-1.37) with DP due to mental diagnoses.

**Table 3:** Hazard ratios (HR) for disability pension due to mental diagnoses, whole twin cohort

**Self-rated health and health behaviors**	**Whole twin cohort (N= 28 613) HR (95% CI)**			
	**Base model**^**1**^	**Model 2**^**2**^	**Model 3**^**3**^	**Full model**^**4**^
**Self-rated health**				
Good	1.00	1.00	1.00	-
Moderate	**2.52 (2.08-3.07)**	**2.50 (2.03-3.07)**	**2.43 (1.97-3.00)**	-
Poor	**6.61 (5.39-8.11)**	**6.34 (5.05-7.95)**	**5.87 (4.65-7.41)**	-
**Leisure-time physical activity**				
High	1.03 (0.79-1.35)	1.03 (0.79-1.35)	1.03 (0.78-1.36)	1.10 (0.83-1.44)
Moderate	1.00	1.00	1.00	1.00
Low	**1.48 (1.18-1.87)**	**1.40 (1.11-1.77)**	1.25 (0.98-1.58)	1.06 (0.84-1.36)
**Body mass index**				
Underweight	**2.45 (1.50-3.98)**	**2.48 (1.52-4.05)**	**2.15 (1.32-3.52)**	**1.88 (1.15-3.06)**
Normal weight	1.00	1.00	1.00	1.00
Overweight	**1.25 (1.05-1.50)**	**1.26 (1.05-1.51)**	**1.28 (1.07-1.54)**	**1.24 (1.03-1.48)**
Obesity	**1.38 (1.03-1.84)**	1.32 (0.99-1.77)	1.27 (0.95-1.71)	1.01 (0.75-1.37)
**Tobacco use**				
Never tried	1.00	1.00		1.00
Former	**1.59 (1.06-2.39)**	**1.58 (1.05-2.37)**	**1.74 (1.16-2.63)**	**1.70 (1.13-2.56)**
Current occasional	1.10 (0.89-1.35)	1.07 (0.87-1.32)	1.09 (0.88-1.35)	1.08 (0.87-1.34)
Current regular	**1.73 (1.41-2.12)**	**1.71 (1.39-2.11)**	**1.73 (1.38-2.16)**	**1.62 (1.29-2.03)**
**Alcohol consumption**				
Abstainer	**2.40 (1.90–3.03)**	**2.31 (1.83–2.93)**	**2.23 (1.76-2.83)**	**1.99 (1.57–2.54)**
Light frequent	1.00	1.00	1.00	1.00
Moderate frequent	1.25 (0.91-1.72)	1.22 (0.89-1.68)	1.11 (0.80-1.53)	1.07 (0.78-1.49)
Heavy frequent	1.12 (0.71-1.75)	1.04 (0.66-1.68)	0.95 (0.60-1.50)	0.98 (0.61-1.54)
Light infrequent	1.29 (0.82-2.04)	1.30 (0.82-2.06)	1.26 (0.79-2.00)	1.09 (0.69-1.73)
Moderate infrequent	1.25 (0.97-1.62)	1.26 (0.97-1.64)	**1.30 (1.00-1.69)**	1.18 (0.91-1.54)
Heavy infrequent	1.27 (0.98-1.66)	1.28 (0.98-1.66)	1.26 (0.97-1.65)	1.20 (0.92-1.57)

1. Adjusted for age (continuous) and sex.

2. Base model + education, marital status and severity of diseases.

3. Model 2 + health behaviors (BMI continuous).

4. Model 3 + SRH.

To account for the familial factors in the associations, the data on discordant twin pairs were analyzed (Table 4). In these analyses, the associations of poor or moderate SRH, under- and overweight/obesity, former use of tobacco and abstention from alcohol with DP were found to remain comparable to those of the base model for the whole twin cohort.

**Table 4:** Hazard ratios (HR) for disability pension due to mental diagnoses, discordant twin pairs

**Self-rated health and health behaviors**	**Discordant twin pairs HR (95% CI)**		
	**Monozygotic &dizygotic**^**1**^** (n=229)**	**Monozygotic**^**2**^** (n=95)**	**Dizygotic**^**3**^** (n=134)**
**Self-rated health**			
Good	1.00	1.00	1.00
Moderate	**1.70 (1.04-2.78)**	1.73 (0.76-3.95)	1.68 (0.91-3.13)
Poor	**5.65 (2.81-11.4)**	**8.21 (2.37-28.5)**	**4.57 (1.95-10.7)**
**Leisure-time physical activity**			
High	1.00 (0.49-2.06)	0.80 (0.21-2.98)	1.23 (0.52-3.01)
Moderate	1.00	1.00	1.00
Low	1.00 (0.54-1.84)	1.00 (0.35-2.85)	1.03 (0.49-2.18)
**Body mass index**			
Underweight	1.88 (0.44-8.01)	1.00 (0.06-15.7)	2.44 (0.43-13.9)
Normal weight	1.00	1.00	1.00
Overweight	**2.48 (1.43-4.30)**	1.98 (0.84-4.64)	**2.96 (1.42-6.15)**
Obesity	2.28 (0.88-5.87)	2.61 (0.38-18.3)	2.25 (0.75-6.74)
**Tobacco use**			
Never tried	1.00	1.00	1.00
Former	1.38 (0.44-4.25)	0.71 (0.13-3.92)	2.17 (0.44-10.7)
Current occasional	0.77 (0.40-1.47)	0.98 (0.35-2.77)	0.70 (0.30-1.65)
Current regular	1.06 (0.53-2.11)	1.10 (0.38-3.17)	1.11 (0.44-2.76)
**Alcohol consumption**			
Abstainer	**2.17 (1.06–4.45)**	1.93 (0.54-6.96)	**2.63 (1.05–6.62)**
Light frequent	1.00	1.00	1.00
Moderate frequent	1.24 (0.53-2.91)	0.46 (0.11-1.87)	2.70 (0.81-9.02)
Heavy frequent	0.48 (0.12-1.90)	**Not enough pairs**	0.46 (0.09-2.36)
Light infrequent	3.67 (0.91-14.8)	2.80 (0.24-32.6)	3.98 (0.71-22.4)
Moderate infrequent	1.03 (0.55-1.93)	0.52 (0.18-1.49)	1.71 (0.75-3.91)
Heavy infrequent	2.10 (0.99-4.46)	1.09 (0.33-3.53)	**3.61 (1.28–10.2)**

^1^Adjusted for age, sex, genetics (50-100%) and shared environment (100%).

^2^Adjusted for age, sex, genetics (100%) and shared environment (100%).

^3^Adjusted for age, sex, genetics (50%) and shared environment (100%).

## Discussion

This prospective population-based twin cohort study revealed that poor or moderate SRH, under- or overweight, former use of tobacco products and abstention from alcohol seem to be independent risk factors for DP with mental diagnoses, even after accounting for various confounders, including familial factors. Thus, expected hypotheses regarding associations between poor/moderate SRH, under- or overweight, tobacco use, and being an abstainer from alcohol and DP were confirmed, but those concerning low physical activity in leisure time, obesity, and frequent consumption of alcohol were not. These findings can be used for early identification of individuals at risk of DP in, for example, health care or the workplace, and for the implementation of health promotion activities in various settings.

The finding that SRH is a strong risk factor for DP due to mental diagnoses is in accordance with the two previous studies of the association between SRH and risk of DP due to mental ill-health [15,18]. This was expected based on the fact that poor SRH is a well documented predictor of morbidity [4,5]. Nevertheless, given the scant attention paid to SRH in relation with DP due to mental diagnoses, there is a need for more studies to confirm this association.

Regularly physical activity in leisure time has shown to be associated with psychological well-being [6], while a sedentary life-style appears to be associated with the development of mental disorders, such as depression and anxiety [6-8]. However, whether a sedentary life-style is directly associated with risk of DP due to mental diagnoses is barely unknown [27]. Previous studies have shown that high leisure-time physical activity is protective for DP [19,21-27]. The results of this study point in the direction that low leisure-time physical activity may add to the risk of DP due to mental diagnoses. However, SRH and other factors shared by twins, such as parental attitudes towards being physical activity or not during childhood, appear to influence this association.

In accordance with three previous studies [30,32,33], under- and overweight were found to be associated with risk of DP due to mental diagnoses, independently of other confounding factors of this study, including familial ones. Under- and overweight are the extremes deviating from normal weight and therefore a U-shaped association could be expected (Neovius et al. 2008a). However, for the association of obesity with DP, SRH appears to be an influential factor. This may indicate that being obese is accompanied by poorer health, which increases the risk of DP due to a mental diagnosis [9].

In the case of use of tobacco products, this study support the previous finding [22] that being a former tobacco user increase the risk of DP. However, when it comes to regular use of tobacco products this study is not supporting a direct association with DP as found by many earlier studies [19,21-23,25,26,36-38]. It seems as if factors shared by the twins, e.g. being exposed to smoking parents during childhood and/or genetic predisposition to nicotine dependence, may partly explain the association found. Still, given the low statistical power in the discordant twin pair analyses the results need to be interpreted with caution.

The finding that being an abstainer from alcohol entails an increased risk of DP due to mental diagnoses is in line with previous studies [39-44]. In addition, the results in this study point in the direction that infrequent alcohol consumption may increase the risk of DP due to a mental diagnosis. A possible explanation for the association of being an abstainer from or an infrequent consumer of alcohol with DP due to mental diagnoes may be that some of these people may be former alcoholics who no longer drink/drink only a little, or who have one or several diseases that prevent them from drinking. However, in contrast with previous studies [39-45], no elevated risk of mental DP was found for heavy frequent consumption of alcohol. Consequently, this study does not support the view that there is a U-shaped relationship between alcohol consumption and DP related to mental ill-health [39,41]. The reasons for the discrepancy in findings may lie in different ways of categorizing alcohol consumption, such as with regard to the setting of the cut-off point between moderate/occasional and heavy/frequent consumption.

### Strengths and limitations

The main strengths of this study were: the use of a large population-based twin cohort of men and women (N=28 613), which made it possible to account for familial confounding in the analyses; the study’s prospective design; the relatively long follow-up (median 10 years); and the use of high quality registry data on DP and other background factors, with no loss to follow-up. An additional strength was access to information on current and/or previous history of MD and/or GAD, which made it possible to study the influence of these disorders at the baseline of this study. MD and GAD are two examples of mental disorders (WHO 2007), which account for the majority of newly granted DPs due to mental diagnoses today [1]. However, even after exclusion of individuals with MD or GAD at baseline from the cohort, the associations of the investigated factors with DP remained. This suggests that mental disorders, as measured by MD and GAD, and health factors and behaviors may co-exist without affecting the associations between SRH, health behaviors and DP due to mental diagnoses. Other strengths were that the self-reported data were gathered using a standardized procedure [61], and the use of validated/well known-instruments [28,49,61,64-66,73]

A limitation of the study was the relatively low statistical power of the analyses of the discordant twin pairs, especially when stratified for zygosity. Hence, the interpretation of these analyses merit caution. However, although the point estimates decreased in the discordant twin pair analyses, the magnitudes and directions of effects remained largely unchanged. This tends to confirm the results for the whole cohort, rather than indicating the presence of familial confounding. Another limitation of the study is that the health factors and behaviors were only measured at one point in time; hence they could have been different both before and after the SALT interview. However, adjustment for several important and known confounders was done and is a strength of this study. Finally, given that the sample only included middle-aged twins born and living in Sweden, the results of this study is not valid for younger adults [1], nor to immigrants [74]. Also, twins may be questionable study subjects because of their somewhat different environments during pregnancy and upbringing compared with singletons [75]. Nevertheless, since the findings of this study are in line with previous studies of unrelated individuals this is believed to be a minor issue.

## Conclusions

To conclude, rating one’s general health as either poor or moderate, being under- or overweight, being a former user of tobacco products, or an abstainer from alcohol seem all to be factors independently associated with the risk of DP due to a mental diagnosis, even after accounting for several confounders of this study, including familial factors.

## Competing interests

The authors declare that there are no competing interests.

## Authors’ contributions

PS was the PI of the study and main responsible for data acquisition and ethical approvals. The study was jointly designed by all authors. ÅS was responsible for the data analyses. Findings were jointly interpreted by all authors. ÅS drafted the initial paper outline and all co-authors contributed to successive drafts. The final manuscript was approved by all authors.

## Pre-publication history

The pre-publication history for this paper can be accessed here:

http://www.biomedcentral.com/1471-2458/13/621/prepub
